# Reversal of Sudden Blindness as a Consequence of Isolated Sphenoid Sinus Mucocele: A Case Report and Literature Review

**DOI:** 10.7759/cureus.62158

**Published:** 2024-06-11

**Authors:** Abdullah Imadeddin Malek, Iyad Hamadi, Mouhannad Fakoury, Tarek Al Salhani

**Affiliations:** 1 Otolaryngology - Head and Neck Surgery, Dubai Health, Dubai, ARE

**Keywords:** sphenoid sinus lesions, sphenoid sinus, optic neuropathy, reduced visual acuity, sphenoid sinus mucocele, otolaryngology-head and neck surgery, general otolaryngology

## Abstract

Mucoceles are locally invasive but benign expansive cystic lesions that can arise within paranasal sinuses. Isolated sphenoid sinus Mucoceles (SSM) are quite rare, comprising less than 1% of all paranasal sinus mucoceles. Due to the critical position and proximity of the sphenoid sinus to vital structures, SSMs can cause a multitude of symptoms and complications. We report a case of a 53-year-old man who presented with sudden vision loss and was found to have an isolated SSM. Following surgical drainage and management of the SSM, the patient had full recovery of visual acuity upon discharge.

## Introduction

Mucoceles are locally invasive but benign expansive cystic lesions that are epithelial-lined and mucous-containing. They can originate within all paranasal sinuses (PNS), but are most commonly found in the frontal sinus followed by the ethmoidal sinuses. Isolated sphenoid sinus mucoceles (SSM) are rare, comprising less than 1% of all paranasal sinus mucoceles [1].

Due to the critical position and proximity of the sphenoid sinus to vital structures such as the cavernous portion of the internal carotid artery, oculomotor nerve (CN III), trochlear nerve (CN IV) and two branches of the trigeminal nerve (CN V), the ophthalmic and maxillary branches, SSMs can cause a multitude of symptoms and complications. Despite being pathologically benign, the tendency of SSM to erode and expand into adjacent tissues and structures in its vicinity poses an increased risk for potential complications that can be irreversible, such as vision loss [2].

The most common presenting symptom of SSM is headache, reported in about 87% of cases. Second to that come the ophthalmic manifestations which have a prevalence of 85%. Ophthalmic manifestations include decreased vision, diplopia, proptosis, visual field defects, and external ophthalmoplegia [3]. Early diagnosis and endoscopic surgery are the cornerstones of managing this condition and preventing the possible complications that may arise due to continuous expansion and pressure symptoms on the surrounding structures. We report a case of isolated SSM that presented to our center with complete vision loss in the left eye that had a full return of vision following successful treatment.

## Case presentation

A 53-year-old male patient presented to our center with sudden vision loss in the left eye that started three days prior. This was associated with unilateral left-sided periocular pain that preceded the vision loss by seven days. The patient gave no history of trauma and had no nasal blockage, epistaxis, fever, nausea, or vomiting. He is a known case of Type 2 diabetes on oral hypoglycemic agents, hypertension on antihypertensives, and ischemic heart disease (IHD) on aspirin and clopidogrel following percutaneous coronary intervention (PCI) seven years prior to presentation. He reported no previous history of nasal disease, nasal surgery, and optic or ocular morbidity. 

On initial physical assessment, the patient had decreased visual acuity in the left eye and relative afferent pupillary defect (RAPD). Fundal examination revealed a normal cup-to-disk ratio and pallor of the disk margins indicating optic nerve compression. Ocular movements were intact with normal range of motion. The patient was reviewed by the ophthalmology team and was discharged from their side and referred to the otolaryngology team. Nasal examination revealed mucosal edema, rhinorrhea, and hypertrophied inferior turbinates. There was no evidence of bleeding or nasal discharge. A nasal endoscopy in the clinic revealed a mass in the sphenoethmoidal recess. 

**Figure 1:**
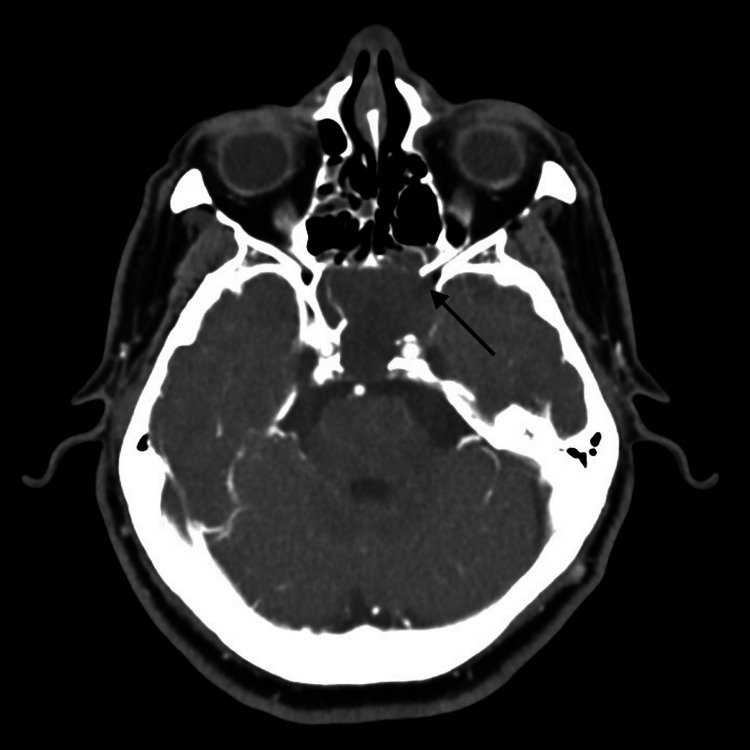
CT scan of the paranasal sinuses with contrast The image shows a heterogenous hypoattenuating mass in the sphenoid sinus, causing erosion of the roof, floor and lateral walls of the sphenoid sinus and encroaching on the ipsilateral optic canal and the superior orbital fissures of the left orbit (black arrow).

**Figure 2:**
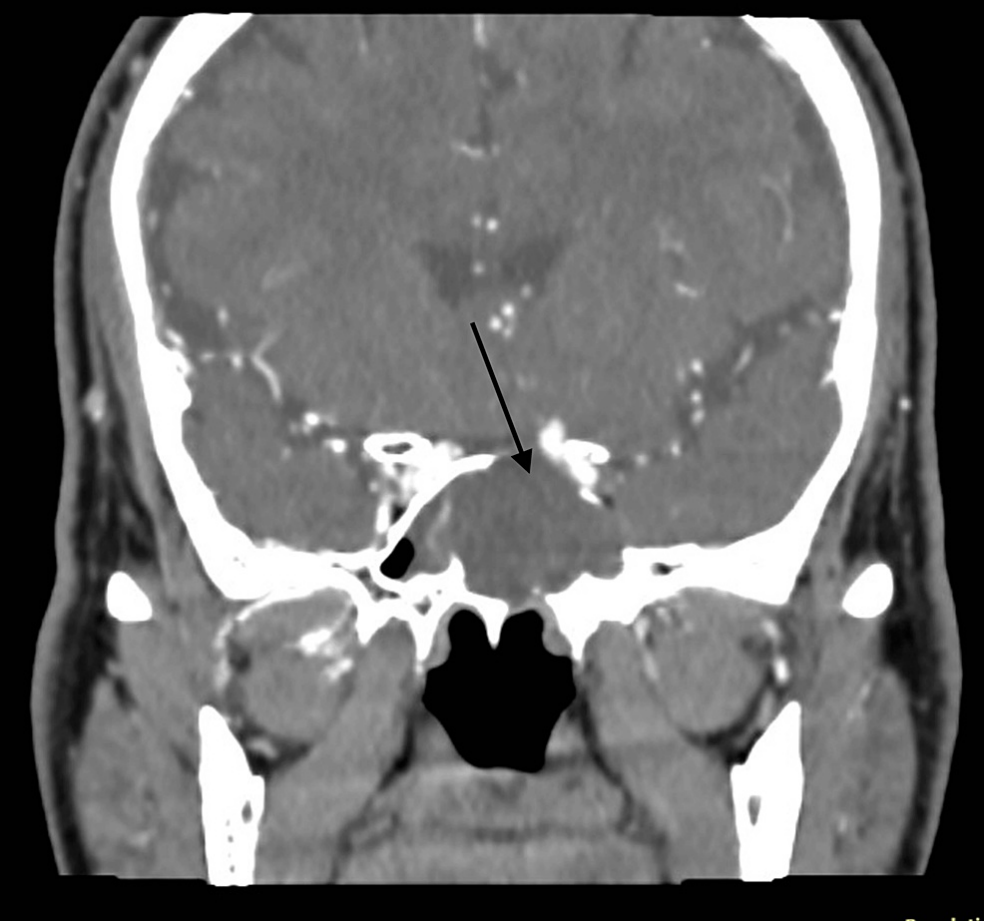
CT scan of the paranasal sinuses in the coronal plane The image shows the same lesion with an erosion of the lateral and superior wall of the sphenoid sinus on the left side, no intracranial involvement can be appreciated (black arrow).

CT orbit and paranasal sinuses with intravenous contrast showed an enlarged sphenoid sinus containing a heterogenous hypo-attenuating mass with no significant enhancement following contrast injection. The mass was causing erosion of the roof, floor and lateral walls of the sphenoid sinus and encroaching on the ipsilateral optic canal and the superior orbital fissures of the left orbit as seen in Figures 1, 2. Although it was intimately related to the brain, there was no invasion of the mass into the brain.

**Figure 3:**
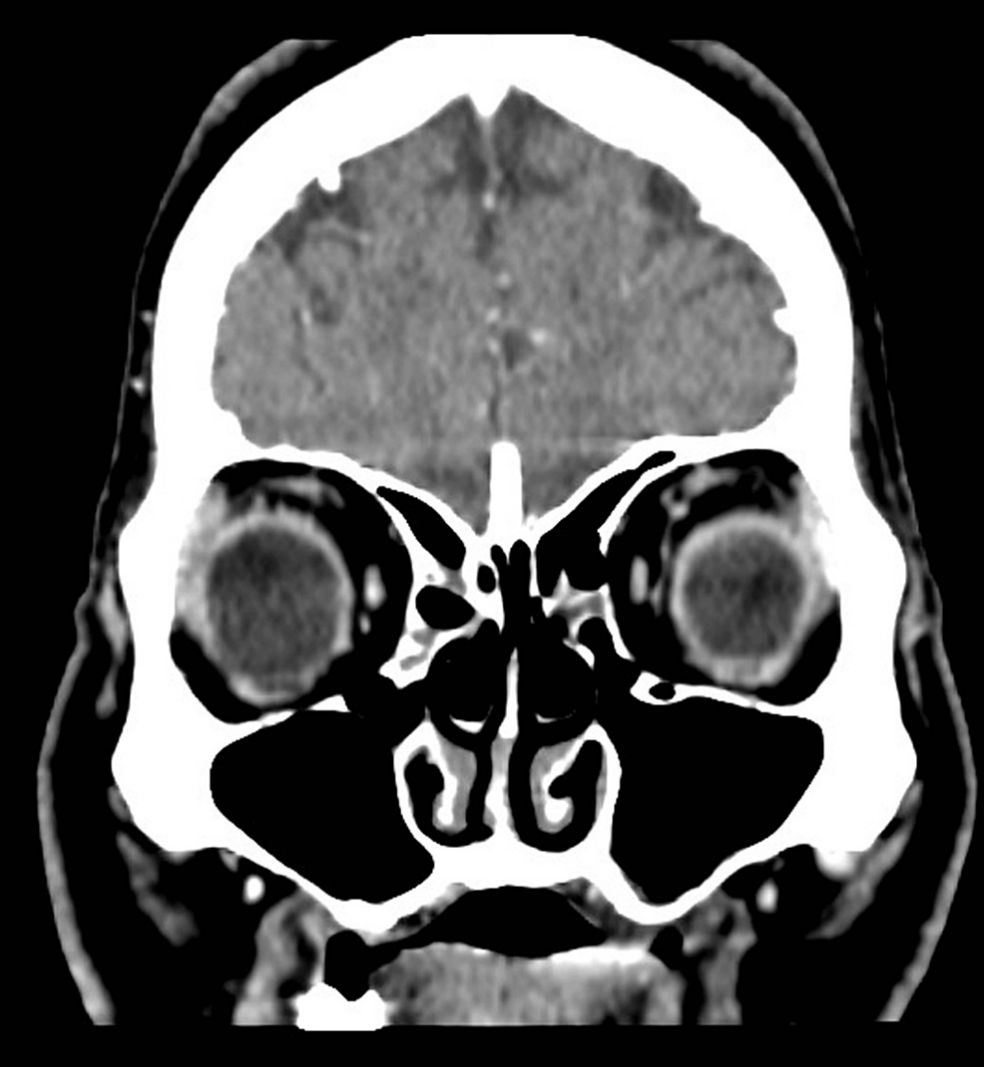
CT scan of the paranasal sinuses with contrast in the coronal plane The image shows clear sinuses including bilateral ethmoidal and maxillary sinuses.

**Figure 4:**
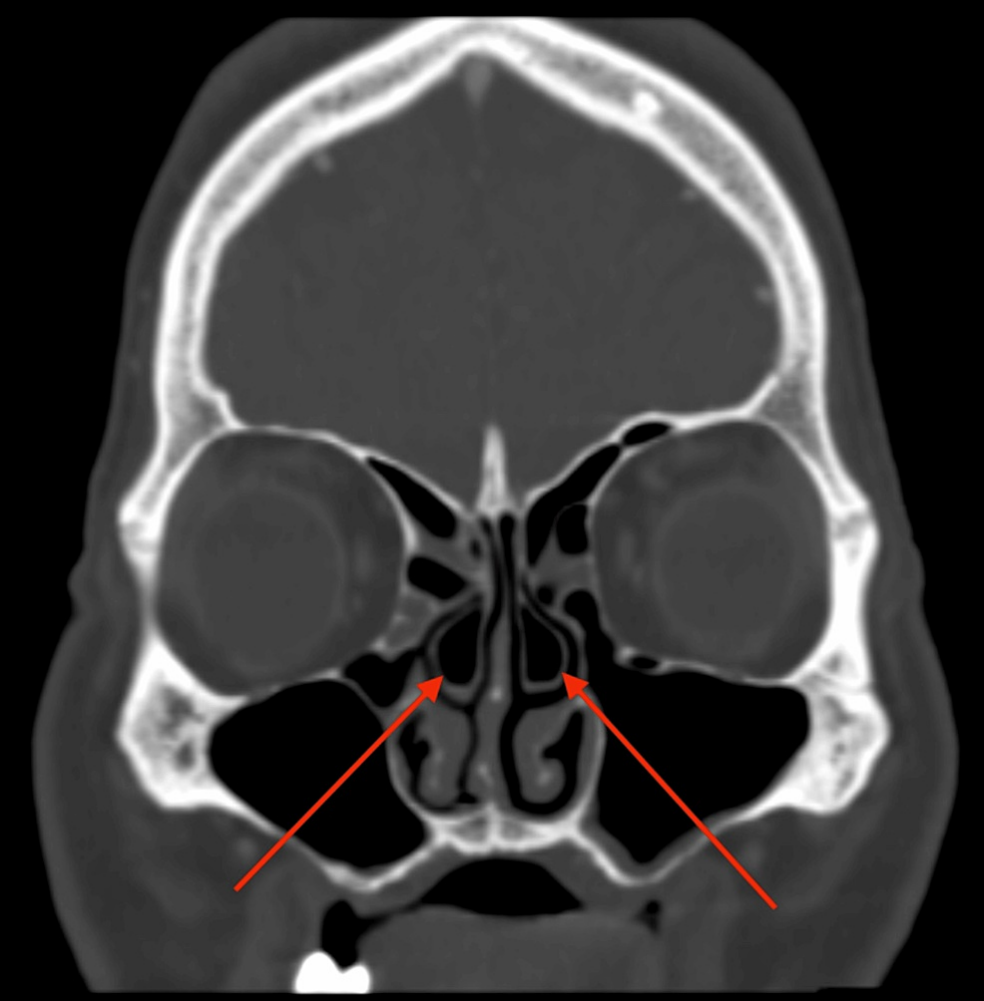
CT scan of the paranasal sinuses in coronal plane in bone window The image shows bilateral concha bullosa (red arrows).

The pituitary gland was not involved. There were no filling defects detected in the cavernous segment of the internal carotid artery. However, the cavernous sinus itself showed reduced enhancement on the left side when compared to the right cavernous sinus. All other sinuses were clear with no detectable pathology as seen in Figure 3. The patient also had bilateral concha bullosa as seen in Figure 4.

Magnetic resonance imaging (MRI) with contrast was recommended for further evaluation which revealed a mucocele in the left sphenoid sinus with left optic nerve compression on the intracanalicular and intracranial cisternal segment of the optic nerve. The patient was taken to the operation theatre for examination under general anaesthesia of the nose and posterior nasal space. A transnasal approach was used to approach the sphenoid sinus as there was no pathology in the ethmoid sinuses that would warrant a transethmoidal approach. Intraoperative findings included mucosal thickening in the sphenoid sinus with thick secretions and a defect in the posterior wall of the sphenoid sinus. A large cystic lesion in the sphenoid sinus extending upwards and encroaching on the superior orbital fissure and the optic nerve was found. The mucocele was opened, draining thick mucoid secretions, and its wall remnants were excised. No pus or infective secretions were appreciated, and as such, no swab was taken from the mucocele contents. A biopsy sample was taken from the sphenoid mucosa and posterior wall of the sphenoid sinus. A cavernous portion of the left carotid artery was exposed as well as the left optic nerve. Duraplasty was not necessary as the dura was intact intraoperatively with no cerebrospinal fluid (CSF) leak. 

The postoperative course showed significant improvement in vision with 50% returning the next day and full recovery of the visual acuity upon discharge two days later. Headaches improved significantly postoperatively. The patient complained of minimal watery discharge from the nose as well as minimal bleeding, which improved on postoperative day 2 and the patient was discharged home. The patient was started on high-dose steroids 60 mg for a duration of seven days postoperatively due to its anti-inflammatory action. Moreover, antibiotics were prescribed and he was advised to avoid any strenuous activity and to return to the emergency department in case of any complaints including visual complaints, bleeding or increased nasal discharge. A follow-up appointment after one week revealed significant improvement in the patient’s clinical condition.

Biopsy samples from the walls of the sphenoid sinus revealed evidence of chronic inflammation as reported by the histopathology report. Samples taken from the posterior wall of the sphenoid sinus showed evidence of fresh and old hemorrhagic fibrinous material as evident by Prussian blue iron stain. Fibrotic sclerotic stromal tissue with a small piece suggestive of granulation-like tissue was also found. No evidence of fungal infection was found as evident by a negative Periodic Acid-Schiff (PAS) stain and Grocott methenamine silver (GMS) stain. No evidence of allergic mucin, eosinophilia, granuloma, or malignancy was appreciated in both samples. A diagnosis of chronic left sphenoidal sinusitis leading to the development of mucocele was made. Follow up after 3 months showed continued improvement with no new complaints or symptoms.

## Discussion

First described in 1889 by John Berg, a Swedish surgeon, isolated Sphenoid sinus mucoceles are considered rare as they constitute only 1-2% of all cases of paranasal sinus mucoceles [1]. On the contrary, frontal sinus mucoceles occur much more commonly, making up more than 50% of all cases of paranasal mucoceles [4]. Although benign in nature, mucoceles can be locally invasive, leading to erosion and destruction of the bony walls of a sinus. Although initially unilateral, SSM typically involves the whole sphenoid sinus at the time of presentation due to its invasive behavior, leading to symptoms bilaterally. Moreover, diagnosis of SSM is often delayed as the symptoms are usually not evident until there is expansion and compression of adjacent structures.

The sphenoid sinus develops within the body of the sphenoid bone, which forms part of the floor of the middle cranial fossa, the orbital apex, and part of the lateral wall of the skull. The superior wall of the sinus is continuous with the roof of the ethmoid sinus and forms part of the anterior and middle floors of the skull base. This means that it is in direct contact with the olfactory nerves, the optic chiasm, and the hypophysis. The anterior wall of the sinus is in contact with the perpendicular plate of the ethmoid and the vomer as well as the lateral ethmoid masses on either side. The sphenoid ostium can be found on the anterior wall of the sinus. The floor of the sinus forms the dome of the choana and the nasopharynx. Lateral walls, which are divided into the anterior orbital area and the posterior cranial area are directly adjacent to important structures such as the internal carotid artery, the optic nerve, and the cavernous sinus. Hence making sphenoid sinus surgeries quite intricate [5].

Regardless of the sinus of origin, the general pathophysiology is believed to be a result of impaired drainage of mucous from the sinus through the ostium. This can be secondary to trauma, a tumor, previous surgery, or a chronic infection. However, other hypotheses exist with regard to the etiology including cystic dilation of glandular structures and cystic development from embryonic epithelial residues [6]. Although no clear etiology of isolated SSM was found in this case, the presence of bilateral concha bullosa can be speculated as a possible cause of sphenoid sinus outflow obstruction and the development of an isolated sphenoid sinusitis causing the formation of an SSM.

Visual impairment is reported in 85% of cases with SSM, second only to headaches, which is considered the most common symptom reported in approximately 87% of SSM cases [3]. The cause of visual impairment is thought to be due to optic nerve ischemia and inflammation caused by displacement of the nerve and compression by the adjacent SSM. Ischemia of the optic nerve is possible due to the mass effect of the mucocele leading to arterial obstruction or venous congestion [2]. Moreover, due to its expansive behavior and its ability to erode adjacent structures, the expansion of SSM can lead to distortion and compression of nearby tissues and even cause bone erosion, allowing it to spread intracranially [7].

Surprisingly, previous research concluded that visual recovery and prognosis are variable following surgical intervention as displayed in Table 1. Some studies have shown that despite prompt diagnosis and early surgery within 24 hours of visual symptoms, there was no improvement in the visual prognosis of the patient. Li et al. concluded no significant correlation exists between the prognosis of visual acuity and early surgical treatment [8]. This was also demonstrated where patients with a history of intervention within two days [9] up to 34 days [10] had complete visual recovery. Another significant case is of a patient with the longest duration of vision loss, four years, prior to surgical intervention who had significant improvement of vision 20/400 to 20/250 following surgical intervention [11]. In addition to that, other factors, including age, sex, and adjuvant intravenous steroids, did not show any significant correlation with full recovery of vision postoperatively. However, most studies have not mentioned any previous ocular comorbidity, and as such, this factor remains an unexplored factor that could have contributed to the aforementioned findings [8].

**Table 1:** Variability of postoperative visual outcome and prognosis with different timeframes from symptom initiation to surgical intervention NLP: No light perception, LP: Light perception

Reference	Age (Years)	Time from first symptoms to intervention	Pre-operative vision	Visual Outcome
Li et al., 2018 [8]	80	11 weeks	Light Perception (LP)	No visual improvement
Li et al., 2018 [8]	76	Eight weeks	Light Perception. (LP)	Improved to 20/400
Li et al., 2018 [8]	13	Within 24 hours	No Light Perception (NLP)	Improved to LP
Nerurkar et al., 2004 [9]	22	Two days	No Light Perception (NLP)	Complete visual recovery
Sharifi et al., 2013 [10]	25	10 days	Light Perception (LP)	Complete visual recovery
Sharifi et al., 2013 [10]	28	34 days	Light Perception (LP)	Complete visual recovery
Sharifi et al., 2013 [10]	12	Two months	N/A	Partial visual recovery
Nakaya et al., 2011 [11]	41	Four years	20/400	Improved to 20/250
Urbinati et al., 2021 [12]	13	Three months	Hand movements only	Complete visual recovery

A similar case to the one we present was reported by Urbinati et al. but in a pediatric 13-year-old patient who complained of progressive loss of visual acuity over a period of three months following which a diagnosis of SSM was made. The patient regained full visual acuity postoperatively and had a complete recovery from his symptoms [12]. Urbinati et al. propose that the cause of this patient’s full recovery is most probably due to his young age, a factor which was previously reported as not significantly correlated to prognosis in patients with SSM [8].

A meta-analysis by Carlson et al. analyzed the predictive factors for vision recovery after optic nerve decompression for chronic compressive neuropathy including sinus mucoceles [13]. In accordance with the findings of Li et al., age was not found to affect post-surgical visual acuity prognosis [8]. On the other hand, the size of the mass compressing the optic nerve showed a statistically significant association of the size of the mass with the likelihood of improvement based on several types of reported data [13]. In contrast to previously mentioned studies that showed no significant correlation between the time of symptoms occurring to the time of surgical intervention [8-11], Carlson et al. suggest that surgery outcomes may be worse if performed more than a year after the diagnosis. Although this data considers all forms of compressive neuropathy, mucoceles were considered in the analysis, and we believe more studies are required to delineate the precise effect of time elapsed from the start of the compressive mucocele symptoms to the time of surgical intervention and the pathophysiological explanation for it.

Carlson et al. established a linear relationship between preoperative visual acuity and postoperative visual acuity which was more noteworthy at the lower and higher end of the vision scale. This translates to significant improvement that can be appreciated in patients with markedly affected visual acuity [13]. Nakaya et al. reported similar findings with a statistically significant linear relationship between pre- and postoperative visual acuity [11].

A study by Chen et al. revealed the possibility of misdiagnosis of optic neuropathy due to sphenoid sinus inflammatory disease, which includes SSM, as optic neuritis, a known manifestation of multiple sclerosis (MS), despite clear differences between the two entities [14]. Differences can be seen in terms of patients’ age, sex, and character of pain associated with both diseases. While optic neuritis and MS are more common in young females, there is no discrepancy in age and sex with regard to patients with SSM. Moreover, there is a significant improvement of pain and marked improvement of visual acuity within three to five weeks in cases of optic neuritis due to MS after initiation of systemic high-dose corticosteroid, a finding not seen with SSM which, in contrast, demonstrates progressive visual acuity deterioration even after initiation of systemic high-dose corticosteroids, showing no response to therapy or only brief improvement. These findings may suggest an atypical form of optic neuritis. As such, the role of radiological imaging prior to initiation of steroid therapy in such cases is emphasized in order to elucidate the cause as either atypical optic neuritis or external compression by adjacent structures such as a sphenoid sinus mucocele [15].

The majority of SSMs contain sterile contents, with a minority being infected with Gram-positive organisms such as *Staphylococcus aureus*, *Staphylococcus epidermidis* and Streptococci. Such infected mucoceles are also known as mucopyoceles which require treatment with antibacterial based on local guidelines and commonly encountered bacteria [16].

Managing sphenoid sinus mucoceles requires surgical intervention. The current gold standard for SSM drainage is using an endonasal endoscopic method, which allows a shorter recovery time, lower incidence of postoperative complications, and a low percentage of recurrence [17,18]. This procedure involves resection of the mucocele by disrupting its walls and aspirating its contents, with the goal being to evacuate the mucocele, relieve the compressive symptoms on adjacent structures, and prevent recurrence in the future [18].

Surgical approaches differ based on anatomical variations or surgeon’s preference. Some authors suggest enlargement of the sphenoidal ostium [19], while others believe this approach may increase the risk of recurrence [20]. While we used a transethmoidal approach to the sphenoid sinus, several other methods can be used to access the sphenoid ostium, including the trans-nasal, trans-septal, and trans-pterygopalatine approach. Although choosing the most appropriate approach can vary on a case-to-case basis as well as the surgeon’s preference, a general algorithm was devised by Elhamsary et al. to guide the surgical approach to benign sphenoid sinus lesions [18]. Surgical approaches and their implications are beyond the scope of this article.

## Conclusions

In conclusion, a high index of suspicion is needed when presented with a case of sudden vision loss and a full workup is imperative to determine any ENT cause of vision loss if the patient is cleared by ophthalmology. Although the rapid intervention was not found to be a prerequisite for visual symptom improvement as previously discussed, we recommend early intervention as soon as a diagnosis of SSM-induced visual symptoms is made. Other factors that come into play and determine the prognosis need to be elucidated and studied further. Moreover, proper documentation and identification of preoperative optic comorbidity is recommended in order to establish any link or patterns that could lead to the variability of outcomes in the future. 
